# Introducing… Solutions for Kids in Pain

**DOI:** 10.1080/24740527.2019.1588641

**Published:** 2019-04-01

**Authors:** Christine T. Chambers

**Affiliations:** Pediatrics & Psychology and Neuroscience, Halifax, Canada

*Are you a clinician who wants to improve pain management for children in your unit or institution? Are you a scientist or trainee who wants to get the results of your pediatric pain research directly into the hands of the people who can use it? Are you a patient or caregiver who wants to learn how you can use your experience to improve pain management for other children and families?*

Canada is a world leader in children’s pain research and effective treatments exist, but this research evidence is not consistently mobilized into practice due to barriers and disjointed efforts. Come to this introductory session to learn more about “Solutions for Kids in Pain” (SKIP), a newly formed knowledge mobilization network, based at Dalhousie University and co-led by Children’s Healthcare Canada. SKIP seeks to bridge the gap between current treatment practices and available evidence-based solutions for children’s pain in Canadian health institutions. SKIP’s vision is healthier Canadians through better pain management for children, with a mission to improve children’s pain management by mobilizing evidence-based solutions through coordination and collaboration. SKIP brings together Canada’s world-renowned pediatric pain research community, front-line knowledge user organizations, and end beneficiaries (patients and caregivers). Guided by a diverse and experienced Board, SKIP capitalizes on the engagement of: 48 Children’s Healthcare Canada member organizations, over 75 partners, 4 regional hubs, and patients and caregivers (using a “Patients Included” approach) to collaborate and co-produce interconnected knowledge mobilization activities. Our goal is improved children’s pain management in Canadian health institutions.

All are welcome. Come and learn more about how *you* can be a part of making a difference for children in pain and their families!

